# Vaginal Douching during the Puerperium

**Published:** 1895-05

**Authors:** 


BUFFALO MEDICAL AND SURGICAL JOURNAL
A MONTHLY REVIEW OF MEDICINE AND SURGERY.

*EDITORS:*

THOMAS LOTHROP, M. D. - - WM. WARREN POTTER, M. D.

All communications, whether of a literary or business nature, should be addressed to the managing editor: 284 FRANKLIN STREET, BUFFALO, N. Y.

VOL. XXXIV. MAY, 1895. No. 10.

### VAGINAL DOUCHING DURING THE PUERPERIUM.

THE question of employing antiseptic vaginal douching as a routine practice during the puerperium was discussed by the American Association of Obstetricians and Gynecologists during its meeting at Toronto in September, 1894, by several eminent obstetricians. From the published reports we present herewith a condensed summary which may prove of interest to our readers, indicating that the pendulum of public opinion is swinging in an opposite direction to that which it took a few years ago; or, at least, obstetricians are less radical on the subject than formerly.

Professor Adam H. Wright, of Toronto, opened the discussion with a paper entitled, Should Antiseptic Vaginal Douching be made a routine practice during the Puerperium? Dr. Wright pointed out the position that Semmelweiss occupied with reference to modern antiseptic midwifery. As early as 1847, Semmelweiss demonstrated the puerperal fever was caused by the introduction of putrescent substances deposited in or about the genital tract of the parturient woman. Here was the first attempt to show that the so-called puerperal fever of the text-books is, in reality, puerperal septicemia. Investigations and experiments by Pasteur and Lister followed, giving additional weight to the doctrine of Semmelweiss and adding much to our knowledge of aseptic and antiseptic surgery and midwifery.

These doctrines were somewhat slow of adoption, but finally spread over the civilized world and mortality rates in the lying-in room were greatly reduced. In 1883, the New York Academy of Medicine held a remarkable discussion on the subject, in which Fordyce Barker and Gaillard Thomas were the chief disputants.

Every obstetrician is familiar with this great controversy. Dr. Wright says the douching wave reached its greatest height about that time, but since then a reaction has set in and now opinions are divided as to the utility of the measure in normal cases. Bacteriologists have taught us much on the subject, but have not yet proved definitely what forms of bacteria cause the poisoning. The important thing, however, from a clinical standpoint, to recognize is that septic matter when introduced from without by dirty finger-tips, dirty instruments and from dirty surroundings of all sorts creates all the mischief. Professor Wright summarizes his opinions under four general heads, as follows :

1. Douching disturbs that perfect rest and quiet which are so desirable for a patient after labor.

2. Douching is unscientific on surgical grounds. After labor the utero-vaginal canal is bruised and wounded. On surgical principles the most important points in the treatment are rest, pressure, position and drainage.

3. Douching does not lessen the dangers accruing from the presence of bacteria in the vagina. This he acknowledges to be the most difficult contention to prove definitely. It is generally agreed, however, that in normal cases the vaginal mucus is strongly acid and is destructive to pathogenic cocci. Vaginal antiseptic injections may interfere with this normal acidity, and thus chemically lessen the resistance of the tissues to bacteria.

4. Douching is actually dangerous. It is apt to disturb clots and thus open avenues for infection ; to open lacerations of the cervix and vagina and thus prevent them from healing ; to wash bacteria into the uterine cavity and thus cause septic metritis.

Dr. Wright closes his admirable paper by stating that he has given his opinions as they now exist, formed after considerable observation and upon much experience, but that he does not declare them as final or unalterable, and that he is as anxious now as ever to learn something new about aseptic and antiseptic methods.

Professor William H. Taylor, of Cincinnati, followed with a short paper entitled, Shall the Vaginal Douche be used after Normal Delivery ? Dr. Taylor, too, has ceased the routine use of the douche in all normal cases, and is content with strenuously using asepsis,—cleanliness of patient, nurse and physician,—together with the application of a sublimated pad to the vulva immediately after delivery, its use to be continued until the danger of the introduction of germs has passed.

Recognizing the chemical reaction of the vaginal mucous membrane as important in determining the necessity of douching, he asserts his belief that the habitual use of test paper may be a safe guide in indicating the use of the douche. In other words, if the reaction is slightly acid, neutral or alkaline, the sublimate douche should be used, otherwise not.

Professor J. Edwin Michael, of Baltimore, continued the discussion under the head, Obstetric Antisepsis. He asserted that, as a rule,—one with only very rare exceptions,—a clean doctor, a clean nurse, a clean patient and cleanly surroundings will give a normal puerperium. He asserts that a normal woman should be allowed to have a normal labor and not be meddled with by even an examining finger, as in such cases palpation gives the necessary information. This is his rule in private practice; in hospital practice, however, where patients are subjected to examinations by students and nurses, he believes that both ante- and post-partum douches are necessary and also employs them after obstetric operations.

The singular unanimity of opinion of these three eminent teachers, all remote from each other and the opinion of each unknown to the other, offers much food for reflection, and we present this imperfect synopsis to our readers for the purpose of stimulating a further expression of opinion on the subject.

This discussion may be found in full in Volume VII. of the transactions of the association referred to.

